# Wide-field choroidal thickness profile in healthy eyes

**DOI:** 10.1038/s41598-018-35640-9

**Published:** 2018-11-21

**Authors:** Mohammed Abdul Rasheed, Sumit Randhir Singh, Alessandro Invernizzi, Carlo Cagini, Abhilash Goud, Niroj Kumar Sahoo, Mariano Cozzi, Marco Lupidi, Jay Chhablani

**Affiliations:** 1 Smt. Kanuri Santhamma Centre for Vitreo-Retinal Diseases, L. V. Prasad Eye Institute, Hyderabad, 34 India; 2 Eye Clinic, Department of Biomedical and Clinical Science “Luigi Sacco”, University of Milan, Milan, Italy; 3 Department of Biochemical and Surgical Sciences, Section of Ophthalmology, University of Perugia, Perugia, Italy

## Abstract

The introduction of wide field optical coherence tomography (WF-OCT) has provided newer insights in the imaging of peripheral choroid. We evaluated choroidal thickness (CT) and large choroidal vessel thickness (LCVT) of 20 eyes in horizontal and vertical meridians using WF-OCT. A high-definition line scan through the fovea in both horizontal and vertical meridian was captured in primary and extremes of gaze to obtain scans up to mid-equator. CT and LCVT measurements were done across predefined points in macular area and all quadrants. LCVT was calculated after identifying a large choroidal vessel near choroidoscleral interface. The main outcome measures were differences in CT and LCVT in macular and four quadrants. Mean CT (331.23 ± 76.34 µ) and LCVT (201.46 ± 54.31 µ) in vertical macular segment were significantly more than CT (245.79 ± 55.38 µ; p = 0.0002) and LCVT (150.48 ± 52.58 µ; p = 0.004) in horizontal macular segment. CT at peripheral points in all quadrants was significantly reduced as compared to subfoveal CT (all p values < 0.05) with maximum reduction in inferior quadrant (64.5%). Using linear regression, only quadrant had a significant effect on CT and LCVT (both p < 0.001). CT and LCVT are highest at the macular area with reduction towards the periphery. The contribution of LCVT to CT is less at the fovea compared to other peripheral points.

## Introduction

The choroid is the most vascular structure of the eye and plays a role in the pathogenesis of various chorioretinal disorders^1^. The detailed analysis of choroid involves the study of multiple quantitative and qualitative parameters including choroidal thickness (CT), volume (CV), large or medium choroidal vessel thickness (LCVT; MCVT), vascularity index (CVI) and contour^2–8^. The advancements in optical coherence tomography (OCT) techniques including enhanced depth imaging (EDI-OCT) and swept source (SS-OCT) during the past decade have facilitated an in depth *in vivo* analysis of choroid^3,9,10^. There is extensive literature describing CT in the subfoveal or macular area in healthy as well as diseased eyes^3,4,7,11,12^. However, the details of peripheral choroid have not been available in diseases affecting the peripheral retina such as central serous chorioretinopathy (CSCR), polypoidal choroidal vasculopathy (PCV), diabetic retinopathy (DR), and pathological myopia^10,13–17^.

The study of peripheral choroidal details is possible with wide-field imaging involving up to 180–200 degree field of view (FOV)^9,10,18,19^. The wide FOV SS-OCT provides a larger field of view of 80 degrees (=20 mm) in the primary gaze or using manual montage to study up to 200 degrees till the equator in both the horizontal and vertical meridians^9,10,18^. This is higher than any other available conventional OCT imaging system. We have earlier studied the wide field CVI in macular and peripheral fundus in all quadrants using line scans and manual montage^20^. The macular CVI was significantly lesser as compared to all quadrants despite having maximum choroidal thickness. The reduced CVI at the macular area could be due to a disproportionate increase in the stromal component at the subfoveal level^20^.

Apart from CT and CVI, a recent interest in thickness of choroidal vessels has expanded our understanding about the choroid^5,21^. Previous reports have shown increased large choroidal vessel thickness and medium choroidal vessel thinning in eyes with CSCR and high myopia respectively^22,23^. Though LCVT was not analyzed separately in our previous publication, the possibility of reduced vascular thickness in the macular area compared to peripheral choroid was considered based on CVI results^20^.

The literature on peripheral choroid using advanced imaging modalities is very limited^9,10,18,19^. In the present study, we aim to study the wide-field choroidal details at different positions across the horizontal and vertical meridians. We report the regional variation in CT and LCVT up to the mid-equator using SS-OCT.

## Results

### Demographic details

The analysis included 20 eyes of 20 healthy subjects. The cohort included 9 males and 11 females with mean age of 28.85 ± 6.29 years (range from 18–42 years). A total of 14 myope, 5 hyperope and 1 emmetrope were included with the mean refractive error (spherical equivalent) of −1.05 ± 1.33 diopters (D) (range −3.00 D to +0.75 D).

### Average CT and LCVT measurements of horizontal and vertical scans

The mean (±SD) CT in horizontal and vertical macular segment was 245.79 ± 55.38 µ and 331.23 ± 76.34 µ respectively which was statistically significant (p = 0.0002). Similarly, mean LCVT (±SD) for horizontal macular segment was 150.48 ± 52.58 µ whereas corresponding value for vertical macular segment was 201.46 ± 54.31 µ (p = 0.004). The mean subfoveal CT (SFCT) and LCVT (SF-LCVT) were 337.44 ± 82.70 µ and 187.91 ± 61.30 µ respectively in horizontal scan whereas the mean subfoveal CT and LCVT in the vertical scan were 353.40 ± 75.35 µ and 205.96 ± 60.36 µ respectively. Both the SFCT and SF-LCVT were thicker in the vertical scan than horizontal but were not statistically significant (p = 0.53 and 0.32 respectively). The corresponding values of CT and LCVT in macular segments and all other quadrants are described in detail in Table 1. Intraclass correlation coefficient (ICC) for measurements showed excellent agreement (ICC = 0.94 to 0.96).

**Table 1:** Shows the mean and standard deviation (SD) of choroidal thickness (CT) and large choroidal vessel thickness (LCVT) in horizontal and vertical meridians involving all locations in macular segments and all four quadrants.

	CT (µ)	LCVT (µ)
**Horizontal Scan (Mean ± SD)**		
**Temporal**	**173**.**09 ± 49**.**85**	**92**.**62 ± 37**.**22**
T2	136.99 ± 47.23 (p < 0.001)*	75.24 ± 30.74 (p < 0.001)
T1	209.19 ± 52.49 (p < 0.001)	110.01 ± 43.72 (p = 0.001)
**Macular**	**245**.**79 ± 55**.**38**	**150**.**48 ± 52**.**58**
TM	285.38 ± 47.04 (p = 0.07)	159.79 ± 38.11 (p = 0.66)
HSF	337.44 ± 82.70	187.91 ± 61.30
NM	114.57 ± 36.39 (p < 0.001)	103.74 ± 58.35 (p < 0.001)
**Nasal**	**169**.**22 ± 56**.**07**	**123**.**05 ± 55**.**18**
N1	146.68 ± 58.62 (p < 0.001)	109.06 ± 45.47 (p = 0.001)
N2	208.43 ± 58.63 (p < 0.001)	148.01 ± 57.48 (p = 0.21)
N3	152.57 ± 50.97 (p < 0.001)	112.10 ± 62.60 (p < 0.001)
**Vertical Scan (Mean ± SD)**		
**Superior**	**142**.**21 ± 49**.**51**	**88**.**25 ± 32**.**83**
S2	107.73 ± 33.29 (p < 0.001)	72.58 ± 19.91 (p < 0.001)
S1	176.70 ± 65.74 (p < 0.001)	103.93 ± 45.75 (p < 0.001)
**Macular**	**331**.**23 ± 76**.**34**	**201**.**46 ± 54**.**31**
SM	356.44 ± 78.38 (p = 1.00)	196.65 ± 47.30 (p = 0.99)
VSF	353.40 ± 75.35	205.96 ± 60.37
IM	283.86 ± 75.29 (p = 0.018)	201.78 ± 55.27 (p = 1.00)
**Inferior**	**126**.**63 ± 57**.**19**	**82**.**46 ± 48**.**03**
I1	163.59 ± 77.05 (p < 0.001)	104.31 ± 62.18 (p < 0.001)
I2	89.68 ± 37.34 (p < 0.001)	60.61 ± 33.89 (p < 0.001)

**Figure 1:**
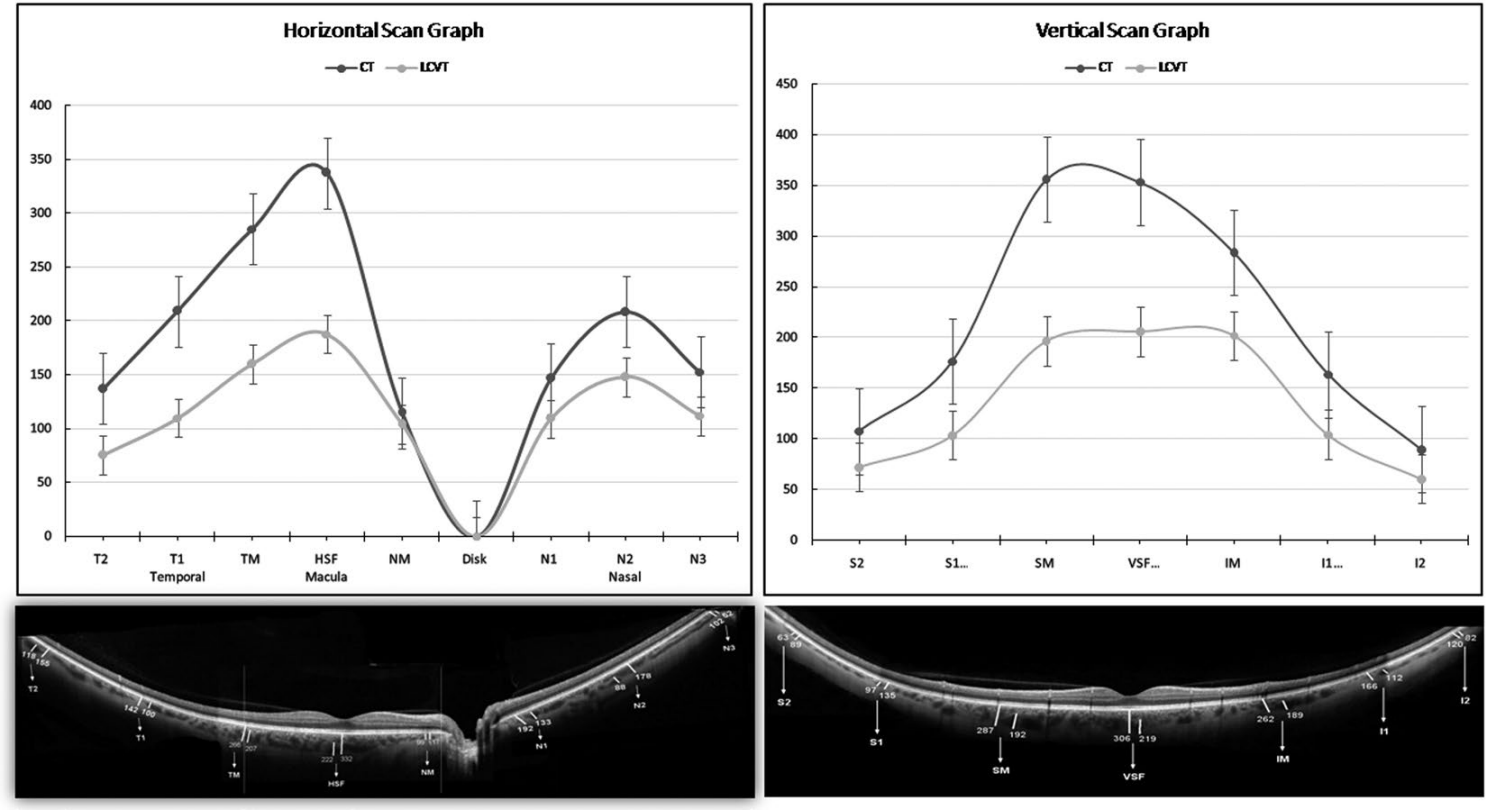
(Top) Shows graphical representation of variation of choroidal thickness (CT) and large choroidal vessel thickness (LCVT). (Bottom) A representative wide-field optical coherence tomography (WF-OCT) at different points in horizontal (left side) and vertical meridian (right side).

*All p values are with respect to subfoveal measurements (either CT or LCVT in respective meridian). HSF, horizontal subfoveal; VSF, vertical subfoveal; TM, NM, SM, IM represent temporal, nasal, superior and inferior macular points respectively. T1, T2, N1, N2, N3, S1, S2, I1 and I2 are points of measurements in respective quadrants as depicted in Fig. 1.

The percentage reduction in CT in different quadrants with respect to subfoveal CT was maximum in inferior quadrant (64.5%) followed by superior (60.1%), nasal (49.8%) and temporal (48.7%) quadrants. Similar to the above findings, percentage reduction in LCVT with respect to SF-LCVT was maximum inferiorly (60.0%) followed by superior (57.1%), temporal (50.7%) and nasal (34.5%) quadrants.

**Figure 2:**
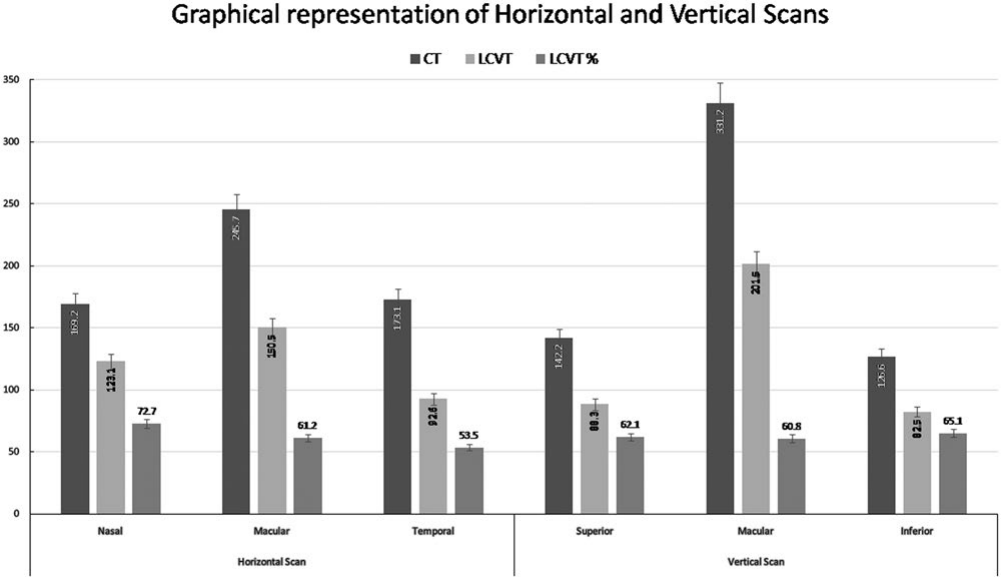
Shows a bar graph comprising mean of choroidal thickness (CT) and large choroidal vascular thickness (LCVT) along with percentage contribution of LCVT in CT in both horizontal and vertical meridian.

In the horizontal scan, the mean LCVT percentage in the macular area and temporal quadrant were 61.2% and 53.5% respectively whereas LCVT percentage in the nasal quadrant was 72.7%. In the vertical scan, the mean LCVT percentage in the superior, macular and inferior quadrants were 62.1%, 60.8%, and 65.1% respectively. Figures 1 and 2 show the graphical representation of changes in CT and LCVT in different segments in both the meridians along with the mean values of CT and LCVT.

### Coefficient of variation (CV)

CV (SD/mean) of the choroidal parameters was calculated in all quadrants. Least CV of CT was noted in macular segments in both the horizontal and vertical meridians (0.23). CV in nasal (0.3), temporal (0.29), superior (0.35) and inferior (0.45) quadrant were comparatively higher with maximum CV in inferior quadrant. The CV of LCVT was minimum in vertical macular segment (0.27) followed by horizontal macular segment (0.35) and other quadrants - nasal (0.45), temporal (0.40), superior (0.37) and inferior (0.58).

**Figure 3:**
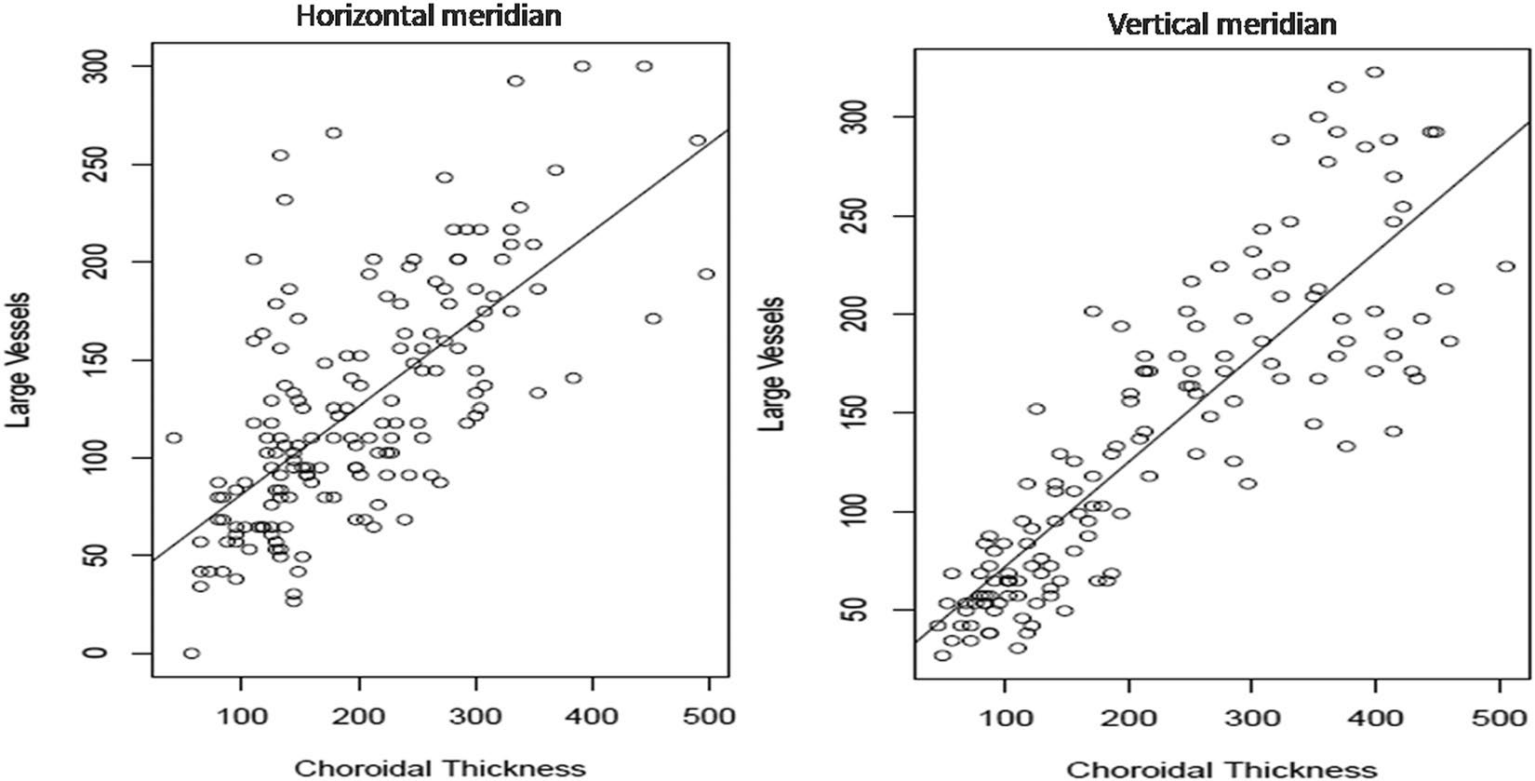
(Left) and (Right) show scatter plots of large choroidal vessel thickness (LCVT; µ) on Y-axis and choroidal thickness (CT; µ) on X-axis in the horizontal and vertical meridian respectively with a statistically significant correlation (both p < 0.001).

The Pearson correlation coefficient between CT and LCVT in the horizontal and vertical meridian was 0.68 and 0.85 respectively suggestive of a significant positive correlation as shown in Fig. 3. Nested analysis of variance (ANOVA) comparing the CT in different regions (total of 8 and 7 points in horizontal and vertical meridian was done. Pairwise comparison of horizontal subfoveal CT and other points using Tukey post hoc test showed that subfoveal CT was significantly higher than all points except temporal macula (p = 0.07). Using Tukey post hoc test, pairwise comparison of vertical subfoveal CT and other points showed SFCT to be significantly higher (p < 0.05) in all but superior macular location (p = 1.00). Similar comparison using nested ANOVA was done for LCVT in both the meridian which showed that SF-LCVT was significantly higher than all nonmacular points (p < 0.05) except N2 point (Table 1).

Using linear regression model, the effects of age, gender, refraction, axial length, intraocular pressure (IOP), quadrant and blood pressure on CT and LCVT were studied. Only quadrant had a significant effect on CT and LCVT (both p-value < 0.001). Rest all variables including age, gender, refraction, axial length, and IOP did not have a significant effect on CT and LCVT. Blood pressure readings were available in only 11 out of 20 eyes. The mean systolic blood pressure (SBP) (115.63 ± 5.39), diastolic blood pressure (DBP) (76.63 ± 4.24), mean arterial pressure (MAP) (89.63 ± 4.05) and mean ocular perfusion pressure (MOPP) (50.06 ± 3.39) mm Hg did not show a significant effect on CT and LCVT.

## Discussion

We studied the mid-equatorial choroidal changes in healthy eyes using wide-field OCT. Our results show that mean CT was maximum in vertical macular segment followed by horizontal macular segment and 4 quadrants (temporal >nasal >superior >inferior). The possible reason for highest SFCT could be the high metabolic demand of central macula which derives its nutrition exclusively from choroid^11^. The macular CT has been studied previously with superior macular subfield showing higher CT compared to SFCT^24^. We also obtained similar results with higher CT in the superior macula (SM) which was not statistically significant. The reduced CT and LCVT in horizontal macular segment compared to vertical macular segment were due to the inclusion of peripapillary area in the horizontal segment where the choroid thinning occurs before its termination at the optic disc.

The gradient of change in CT in horizontal meridian was gradual compared to the vertical meridian where it was much steeper. The inferior quadrant was noted to have minimum CT and LCVT. The percentage reduction in CT and LCVT was maximum in the inferior quadrant (>60%) followed by superior quadrant (>55%). This could be explained by the physiological changes due to effect of gravity. The blood flow in vertical meridian is against gravity in superior quadrant while in the inferior quadrant the flow is with the gravity. This is in contrast to the horizontal meridian where the effect of gravity may not be significantly different at all points. This could explain the wide CT and LCVT difference in vertical meridian. Another reason for the reduced CT in the inferior quadrant could be due to the effect of closure of choroidal fissure which generally occurs in inferior or inferonasal location^25^.

We observed that LCVT changes are not proportionate to the changes in CT from macular region to the periphery. The CT in macular region appears to be maximum, however, significant contribution to CT appears from medium choroidal vessel and choriocapillaris rather than large choroidal vessels. As a corollary, large choroidal vessels contribute significantly to CT toward the periphery. This may partly explain the reason for ocular pathologies to have a differential propensity to macula or peripheral retina. For instance, changes in age related macular degeneration (AMD) are more concentrated in the macular region and is characterized by atrophy of medium and smaller vessel at macula while the peripheral region may be undisturbed^5,12^.

Although all three layers are thickest at posterior pole, the high density of medium choroidal vessels and choriocapillaris at fovea as compared to other peripheral points where the contribution of LCVT to CT is particularly higher needs further attention. This extensive network of smaller vessels subfoveally provide the majority of blood supply to the fovea and this variation may account for the specialized role of fovea in maintaining visual acuity^5^. The analysis of CV of CT and LCVT shows maximum consistency in the macular segment as compared to other quadrants. The inferior quadrant was noted to have maximum variation of CT and LCVT.

Analysis of the watershed zones plays a significant role in understanding the distribution of the choroidal vasculature. This has been made possible by the pioneering work by Hayreh. However, the distribution patterns of these segmental watershed zones have a high interindividual variation thereby precluding any generalized pattern in the normal population^25^. The watershed zone is present at or near the disc in majority of the eyes. This explains the wide variability and reduced CT and LCVT in the peripapillary region.

The study has certain limitations due to its small sample size and cross-sectional nature. The effect of age, gender and axial length on CT and LCVT are difficult to comment due to small numbers in each category. The scans included only line scans in horizontal and vertical meridians, volumetric evaluation of region may help in better understanding of the choroidal vascular network and thickness analysis. The involvement of only a single observer could have added a certain amount of bias, however, the intraobserver reproducibility was excellent (ICC = 0.94 to 0.96). Extrapolation of these findings of the normal eyes to the diseased eyes may be difficult. The presence of mirror artifact in the retinal periphery due to alterations in globe contour limits the image acquisition beyond a certain extent.

In conclusion, this study provides an understanding of the choroidal architecture up to the mid-equator along with the details about the regional differences in the CT and LCVT in healthy eyes. Choroid is thickest at fovea and thickness reduces towards periphery. The CT and LCVT appear to more consistent at macular segments, however, LCVT does not contribute significantly to SFCT compared to the peripheral points. Future studies about variation of these choroidal parameters using wide-field OCT imaging in different chorioretinal diseases could provide detailed information about the disease-specific changes.

### Methodology

The study undertaken was an observational, cross-sectional study involving only healthy subjects. The study was conducted at the Eye Clinic, Sacco Hospital (Milan, Italy) and at the Eye Clinic, S. Maria Della Misericordia Hospital (Perugia, Italy) after prior approval from the ethics committee of the respective institutes. All the study procedures adhered to the tenets of the declaration of Helsinki. A written, informed consent detailing the nature of the study and the risks involved was obtained from all the study participants.

The study included healthy individuals of more than 18 years of age with best-corrected visual acuity (BCVA) of <0.09 logarithm of minimum angle of resolution (logMAR) (Snellen acuity >20/25). The refractive error (spherical equivalent) in the study subjects was ±≤3 diopter (D) with no ongoing or prior history of ocular or systemic disease known to affect retinal or choroidal morphology or physiology.

A comprehensive ocular examination including refraction, BCVA, slit lamp biomicroscopy, IOP, dilated fundus examination using indirect ophthalmoscopy and +90D noncontact lens along with axial length measurements was undertaken to rule out any occult ocular pathology. Systemic examination included a general physical examination and measurement of SBP and DBP. MAP was derived from SBP and DBP (=2/3 of DBP +1/3 of SBP). MOPP was derived from MAP and IOP i.e. 2/3 of difference between MAP and IOP.

### Choroidal Imaging

The OCT scans were obtained using Spectralis® HRA + OCT (Heidelberg Engineering, Heidelberg, Germany) in automated real-time mode at 100 frames/scan. A line scan of high resolution (quality index [QI] more than 30 dB) passing through the fovea in primary gaze was chosen in both horizontal and vertical meridian for detailed analysis. Separate line scans were taken in different directions of gaze involving all quadrants. The images were then manually montaged after removing any overlapped areas to obtain a composite image. The choroidal imaging was done during morning hours (9 am to 12 pm) to avoid any diurnal fluctuation in choroidal parameters.

### Measurement of CT and LCVT

**Figure 4:**
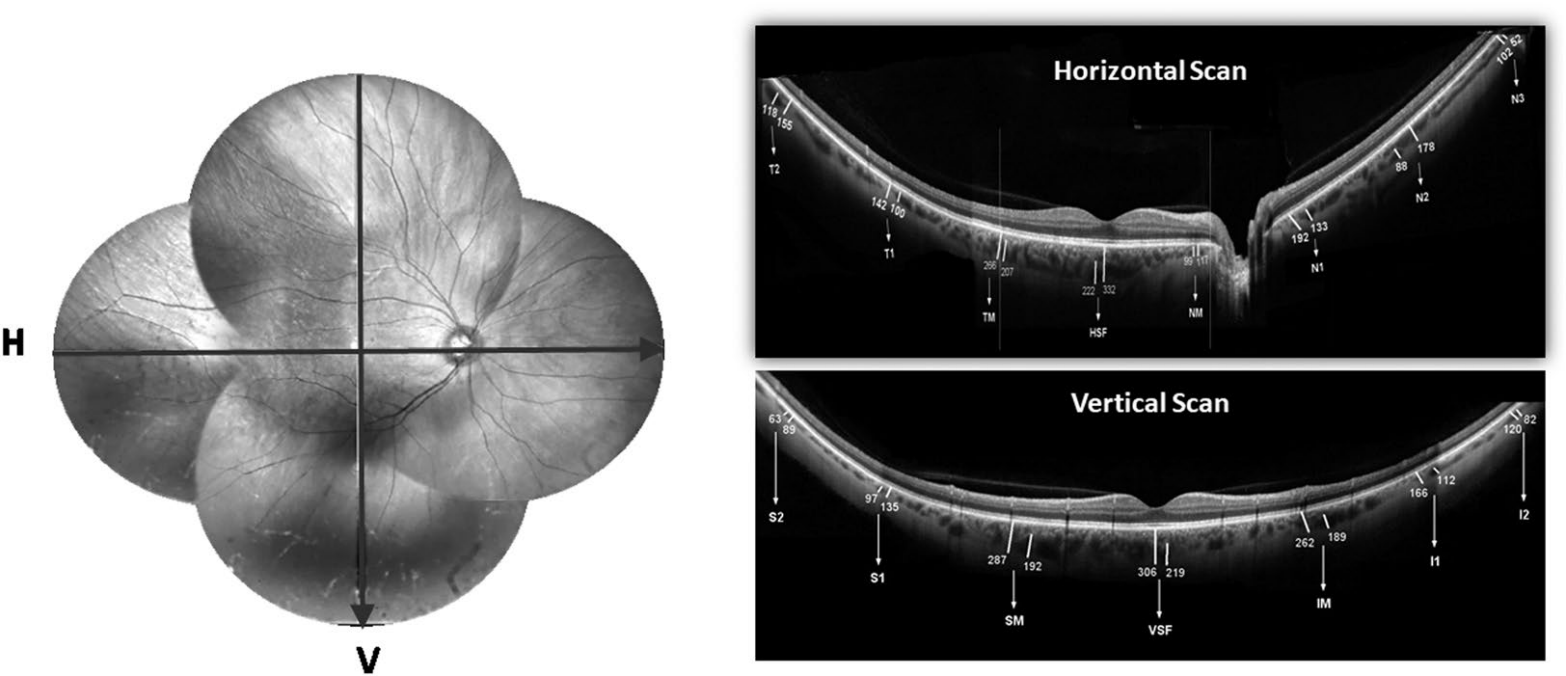
Shows a representative fundus photograph (montage) of right eye with arrows showing the direction of line scans across both horizontal and vertical meridian (left side). Top-right and top-bottom shows representative wide-field optical coherence tomography (WF-OCT) in horizontal and vertical meridian respectively showing the choroidal thickness (CT) and large vessel choroidal thickness (LCVT) in macular segments and all four quadrants. HSF, horizontal subfoveal; VSF, vertical subfoveal; TM, NM, SM, IM represent temporal, nasal, superior and inferior macular points respectively. T1, T2, N1, N2, N3, S1, S2, I1 and I2 are points of measurements in respective quadrants.

Using previously reported automated algorithm, outer and inner choroidal boundaries were delineated^26^. CT was defined as the perpendicular distance between the hyperreflective lines of Bruch’s membrane and choroidoscleral interface (CSI). LCVT was measured at the same or any point within close proximity of the point of measurement of CT if a large choroidal vessel (≥100 µ) was present near CSI. The arbitrary cut-off of 100 µ was chosen as per a previous publication in the absence of any well defined criteria for large choroidal vessel^27^. The unit of measurement was defined as the distance between the temporal margin of the optic disc and center of fovea as described in our previous and other publications^10,20^. Macular segment was defined as twice the disc-fovea distance in both the horizontal and vertical meridian. The respective quadrants were described similarly as 2x disc-fovea distance in each meridian. CT and LCVT were measured at each unit distance from the disk margin in both directions in horizontal and from fovea in the vertical direction. CT and LCVT measurements were done in three areas separately in the horizontal (macular, nasal and temporal quadrants) and vertical scans (macular, superior and inferior quadrants). In view of the absence of choroidal tissue at the site of the optic disc, this area was not considered for any measurements. A total of 8 and 7 points were assessed in the horizontal and vertical scan respectively as depicted in Fig. 4.

The LCVT percentage was measured by dividing the mean LCVT with CT and multiplying the outcome by 100 (LCVT/CTx100). All the measurements were done by a single masked observer (MAR) and repeated twice for 3 prefixed points in both horizontal and vertical meridian after a gap of one week to eliminate any potential source of error. Intraclass correlation coefficient (ICC) was calculated to measure intraobserver reproducibility.

### Statistical analysis

All the measured variables were tabulated and expressed as mean with standard deviation (mean ± SD). LCVT was also expressed as percentage of CT. Nested ANOVA test was done to study variation of CT and LCVT in different segments along with the pairwise comparisons if differences were statistically significant. Pearson correlation was used to study the relationship between CT and LCVT in both the meridians. We also studied the effect of age, gender, refraction, axial length, IOP, quadrant and blood pressure on CT and LCVT using linear regression model. P value of ≤0.05 was considered statistically significant. The statistical analysis was done using R Statistic Software (R version 3.3.1 - The R Foundation for Statistical Computing).

The datasets generated and analyzed during the current study are available from the corresponding author on reasonable request.
